# Polychlorinated Biphenyl 138 Induces Toxicant-Associated Steatohepatitis via Hepatic Iron Overload and Adipose Inflammation

**DOI:** 10.3390/toxics13110932

**Published:** 2025-10-30

**Authors:** Hyeon Jeong Hwang, Mi Hwa Lee, Seung Hui Lee, Byung-Jun Sung, Joong-Gook Kim, Dae Yun Seo, Dae Young Hur, Young Hyun Yoo, JaeHun Cheong, Hye Young Kim

**Affiliations:** 1 Department of Anatomy, Inje University College of Medicine, Busan 47392, Republic of Korea; hhj8965@inje.ac.kr (H.J.H.); hero15p@nate.com (M.H.L.); leesh2550@naver.com (S.H.L.); sungbyungjun@gmail.com (B.-J.S.); sdy925@gmail.com (D.Y.S.); dyhur@inje.ac.kr (D.Y.H.); 2 Department of Molecular Biology, Pusan National University, Busan 46241, Republic of Korea; 3 Research Center, Dongnam Intitute of Radiological Sciences (DIRAMS), Busan 46033, Republic of Korea; jgkim@dirams.re.kr; 4 Department of Anatomy and Cell Biology, Dong-A University College of Medicine, Busan 49201, Republic of Korea; yhyoo@dau.ac.kr

**Keywords:** PCB138, TASH, iron–adipose axis, hepcidin, ferroportin, adipose inflammation, environmental hepatotoxicity

## Abstract

Toxicant-associated steatohepatitis (TASH) is caused by environmental toxicants rather than metabolic factors; however, its pathogenic mechanisms remain poorly understood. Polychlorinated biphenyl 138 (PCB138), a persistent lipophilic contaminant that bioaccumulates in adipose tissue, may promote TASH through unclear mechanisms. In this study, we investigated whether PCB138 induces liver injury via hepatic iron dysregulation and adipose-liver inflammatory signaling. Male C57BL/6 mice received intraperitoneal PCB138 (1, 5, 10, or 50 mg/kg, four injections over six weeks). HepG2 hepatocytes were treated with PCB138 with or without ferric ammonium citrate (FAC), and PCB138-exposed 3T3-L1 adipocytes were co-cultured with HepG2 cells using a Transwell system. PCB138 dose-dependently increased serum transaminase and hepatic non-heme iron levels, with *Hamp* upregulation, macrophage infiltration, and fibrosis. In HepG2 cells, PCB138 synergized with FAC to elevate intracellular Fe^2+^, induced *Hamp*, suppressed *Slc40a1*, and upregulated inflammatory/profibrotic genes. In Transwell co-cultures, TNF-α, IL-6, and IL-1β from PCB138-exposed adipocytes amplified hepatic iron dysregulation and fibrotic responses. These findings demonstrated that PCB138 induced TASH through hepatic iron dysregulation and adipose-derived inflammatory signaling, independent of steatosis. These results highlighted the iron–adipose axis as a novel mechanistic link between PCB138 exposure and liver injury, offering potential therapeutic targets.

## 1. Introduction

Steatohepatitis is a leading cause of liver-related morbidity and mortality worldwide and is increasingly recognized as a precursor to hepatocellular carcinoma. While metabolic dysfunction-associated steatotic liver disease (MASLD) [1,2,3] has been extensively studied, toxicant-associated steatohepatitis (TASH) has recently emerged as a distinct clinical entity [4]. TASH is characterized by lobular inflammation and fibrosis in nonobese, nondrinking individuals with documented exposure to environmental or industrial toxicants, including vinyl chloride and polychlorinated biphenyls (PCBs) [5,6]. Multiple clinical and epidemiological reports indicate that TASH can manifest with minimal or absent steatosis, distinguishing it from MASLD and suggesting fundamentally different pathogenic mechanisms. Although its prevalence remains undefined due to underdiagnosis and limited surveillance, TASH is increasingly reported in exposed populations.

PCBs persist in human tissues decades after the ban in the 1970s [7]. Owing to their lipophilicity and resistance to biodegradation, PCBs bioaccumulate in adipose tissue and biomagnify through the food chain. Among them, PCB138 (2,2′,3,4,4′,5′-hexachlorobiphenyl) is a prevalent non-dioxin-like congener frequently detected in human serum at 20–200 ng/g lipid [8,9,10]. It constitutes a major fraction of dietary PCB exposure through contaminated fish, dairy products, and meat. Epidemiological studies have linked PCB138 exposure to metabolic dysfunction and hepatotoxicity; however, direct clinical evidence linking PCB138 specifically to TASH is lacking [11,12]. This knowledge gap underscores the critical need for mechanistic studies. Unlike dioxin-like congeners that activate the aryl hydrocarbon receptor (AhR), PCB138 acts through non-AhR pathways, such as constitutive androstane receptor activation and oxidative stress induction. Given its persistence and prevalence, elucidating PCB138’s role in TASH pathogenesis is essential for environmental health and risk assessment [13].

Iron homeostasis is a critical determinant of liver health [14,15]. Environmental toxicants can disrupt this balance through hepcidin dysregulation and ferroportin impairment [16], thereby promoting hepatic iron accumulation, which amplifies oxidative stress and contributes to cell death processes [17,18]. Epidemiological studies reporting elevated serum ferritin and transferrin saturation in populations exposed to industrial chemicals support the clinical relevance of iron dysregulation in environmental liver disease.

In parallel, lipophilic toxicants that accumulate in adipose tissue stimulate the release of pro-inflammatory cytokines, including TNF-α, IL-6, and IL-1β. These signals propagate systemic inflammation, impair hepatic metabolic function, and induce hepcidin expression [19]. Emerging evidence suggests crosstalk between iron metabolism and adipose inflammation, where inflammatory cytokines upregulate hepcidin, while iron accumulation enhances inflammatory signaling [20,21,22]. Together, these processes potentially generate a self-amplifying cycle during hepatic injury. However, the specific interaction between iron dysregulation and adipose inflammation during PCB138 exposure remains unclear.

Based on this rationale, we propose the iron–adipose axis as a mechanistic framework for TASH [23]. This framework emphasizes iron metabolism as a central mediator of toxicant-induced injury, extending traditional adipose–liver crosstalk [18,21,24]. This framework extends beyond traditional adipose-liver crosstalk, which primarily focuses on metabolic and inflammatory signaling [25,26], by specifically emphasizing iron metabolism as a central mediator of toxicant-induced injury. Unlike previous models, this axis integrates iron handling into the adipose-liver paradigm, providing a mechanistic explanation for steatosis-independent toxicant injury [27]. While adipose-liver crosstalk broadly encompasses lipid metabolism and cytokine signaling, the iron–adipose axis uniquely focuses on how iron accumulation and adipose inflammation create bidirectional amplification: hepatic iron enhances adipose inflammatory responses, whereas adipose cytokines exacerbate iron dysregulation [28]. We hypothesized that PCB138 induces TASH through steatosis-independent mechanisms involving: (i) disruption of the hepcidin-ferroportin axis, leading to hepatic iron accumulation, (ii) release of adipose-derived inflammatory mediators, and (iii) synergistic amplification between these pathways [29,30].

The objectives of this study were to: (i) characterize PCB138-induced hepatic injury patterns using dose–response analysis in murine models, (ii) elucidate mechanisms of iron homeostasis disruption at transcriptional and post-translational levels, (iii) define adipose-hepatocyte inflammatory crosstalk using co-culture systems [31], and (iv) investigate the iron–adipose axis as a mechanistic framework for TASH pathogenesis. Although murine models and in vitro systems have inherent limitations in recapitulating human disease, they provide controlled conditions for mechanistic investigations.

Understanding these mechanisms has important implications for environmental health assessment. Components of the iron–adipose axis—including serum hepcidin, ferritin, transferrin saturation, and adipose-derived cytokines—could potentially be used as biomarkers for TASH detection and progression monitoring in at-risk populations [15,17,27,31,32]. However, further validation in human cohorts will be required before clinical application. This study will contribute to evidence-based risk assessment frameworks for persistent organic pollutants and inform biomonitoring strategies for communities exposed to legacy contaminants [33].

## 2. Materials and Methods

### 2.1. Chemicals and Reagents

PCB138 (2,2′,3,4,4′,5′-hexachlorobiphenyl; CAS No. 35065-28-2) was purchased from AccuStandard Inc. (New Haven, CT, USA; Cat. No. C-138N; ≥99.8% purity, GC verified). This compound is a Certified Reference Material containing a single analyte, and no other PCB congeners or detectable impurities were reported by the manufacturer or observed in our internal GC–MS verification. The stock solution was prepared in DMSO (30 mM) and stored at −20 °C, and working concentrations were diluted to 30 µM in culture medium (0.1% DMSO as vehicle). Ferric ammonium citrate (FAC; Sigma-Aldrich, St. Louis, MO, USA) was used at 200 µg/mL. FerroOrange (Goryo Chemical, Sapporo, Japan) was used for Fe^2+^ imaging, and Hoechst 33342 (Thermo Fisher Scientific, Waltham, MA, USA) for nuclear counterstaining. Hepatic iron concentrations in mice and hepatocytes were measured using an iron colorimetric assay kit (BioVision, Milpitas, CA, USA).

### 2.2. Animal Studies

All animal procedures were approved by the Committee on Animal Investigations at Dong-A University. Male C57BL/6J mice (8 weeks old, n = 4 per group) were purchased from Samtako Inc. (Osan, Republic of Korea). Animals were housed in a pathogen-free facility under controlled environmental conditions (22 ± 2 °C, 50 ± 10% humidity, 12 h light/dark cycle) with free access to water and a standard chow diet. At 12 weeks of age, mice were randomly assigned to mock (PBS, intraperitoneal), vehicle (corn oil, intraperitoneal), or PCB138 treatment groups (1, 5, 10, or 50 mg/kg, intraperitoneal). PCB138 was dissolved in corn oil and administered four times over six weeks. Following an overnight fast, blood and liver tissues were collected for subsequent analyses.

### 2.3. Cell Culture and Treatments

3T3-L1 mouse embryonic fibroblasts were obtained from the American Type Culture Collection (ATCC, Manassas, VA, USA). Cells were cultured in Dulbecco’s Modified Eagle’s Medium (DMEM; Bandio, Seoul, Republic of Korea) supplemented with 10% fetal calf serum (FCS; Gibco-BRL, Gaithersburg, MD, USA) and 1% penicillin/streptomycin (PS; Bandio, Seoul, Republic of Korea). Cultures were maintained at 37 °C in a humidified atmosphere containing 5% CO_2_. Confluent 3T3-L1 cells were induced to differentiate into adipocytes using DMI induction medium (DMEM containing 10% FBS, 0.05 mM 3-isobutyl-1-methylxanthine (IBMX; Sigma-Aldrich, St. Louis, MO, USA), 0.5 μM dexamethasone (Sigma-Aldrich, St. Louis, MO, USA), and 1 μg/mL insulin (Sigma-Aldrich, St. Louis, MO, USA) for two days, followed by DMII medium (DMEM containing 10% FBS and 1 μg/mL insulin) for two days. Cells were then maintained in DMEM with 10% FBS for four days, with medium changes every two days. To examine the effects of PCB138 on adipocyte differentiation, preadipocytes were incubated with PCB138 (30 µM) during the DMI induction period. HepG2 cells were obtained from ATCC and maintained in DMEM supplemented with 10% heat-inactivated fetal bovine serum (FBS) and 1% (*v*/*v*) PS at 37 °C in a humidified atmosphere containing 5% CO_2_.

### 2.4. Transwell Co-Culture

3T3-L1 preadipocytes were differentiated into adipocytes by sequential treatment with DMI and DMII, as described above. On day 8 of differentiation, mature adipocytes in Transwell inserts (0.4 µm pore size, SPLInsert™, SPL Life Sciences, Pocheon, Republic of Korea) were placed above HepG2 cells cultured in 6-well plates. At this time, co-cultures were treated for 24 h with vehicle (0.1% DMSO), PCB138 (30 µM), FAC (200 µg/mL), or a combination of PCB138 (30 µM) and FAC (200 µg/mL). In adipocyte-only PCB138 cells, PCB138 exposure was restricted to the upper chamber. After 24 h of treatment, HepG2 and 3T3-L1 adipocytes were harvested separately for RNA extraction, protein analysis, and fluorescence imaging.

### 2.5. Histology

Liver and adipose tissues were fixed in 4% paraformaldehyde (24 h), embedded in paraffin, and sectioned at 4 µm. H&E staining was performed to evaluate general morphology and hepatocellular injury. Hepatic iron content was determined using a Perl’s Prussian blue staining kit (Polysciences, Warrington, PA, USA), and collagen deposition in liver sections was evaluated using a Picrosirius Red staining kit (Polysciences). Histological images were obtained and analyzed using a Pannoramic MIDI-II Digital Scanner (3D Histech, Budapest, Hungary). Lipid droplet quantification in liver sections was performed using ImageJ software version 1.54g (National Institute of Health, Bethesda, MD, USA), which analyzes the color and shape of the H&E-stained images to determine the percentage of lipid droplets in the whole field.

### 2.6. Staining and Imaging

For live-cell Fe^2+^ detection, HepG2 cells were incubated with FerroOrange (1 µM, 30 min, 37 °C, protected from light), and nuclei were counterstained with Hoechst 33342 (1 µg/mL, 10 min). Images were acquired using an IRIS Digital Cell Imaging System (BioTek Cytation-based) and analyzed using ImageJ version 1.54g with consistent threshold settings across conditions.

### 2.7. Immunohistochemistry

For immunohistochemical staining of liver and adipose tissues, sections were deparaffinized and subjected to antigen retrieval in citrate buffer (pH 6.0; Abcam, Cambridge, UK). Primary antibodies diluted in Antibody Diluent (Dako, Glostrup, Denmark) were applied overnight at 4 °C: anti-F4/80 (Abcam, Cambridge, UK), anti-MCP-1 (Abcam, Cambridge, UK), anti-TNFα (Proteintech, Rosemont, IL, USA), and anti-IL-6 (CUSABIO, Houston, TX, USA). Immunoreactivity was visualized using the REAL EnVision Detection System, Peroxidase/DAB+, Rabbit/Mouse (Agilent Technologies, Santa Clara, CA, USA), followed by counterstaining with Carrazi hematoxylin (FUJIFILM, Osaka, Japan). Negative control sections were processed under identical conditions with buffer substituted for the primary antibody. Images were acquired using a NanoZoomer Virtual Microscope (Hamamatsu Photonics, Hamamatsu, Japan) and analyzed using NDP.view 2.9.29 RUO software (Hamamatsu Photonics, Hamamatsu, Japan).

### 2.8. RNA Isolation and Quantitative Real-Time PCR Analysis

Total RNA was extracted from cells and liver tissues using TRIzol™ Reagent (Invitrogen, Carlsbad, CA, USA) according to the manufacturer’s instructions. RNA concentration and purity were measured using a NanoDrop 2000 spectrophotometer (Thermo Fisher Scientific). Three micrograms of RNA were reverse transcribed into cDNA using the ZV White cDNA Syn dT15 Master Mix (ZENVIA, Busan, Republic of Korea). Quantitative PCR was performed in a 96-well Real-Time Quantitative Thermal Block (Bioneer, Daejeon, Republic of Korea) using ZV GREEN SY qPCR Master Mix (ZENVIA, Busan, Republic of Korea). Relative gene expression was calculated using the 2^−ΔΔCt^ method with GAPDH as the reference gene. Primer sequences are listed in Supplementary Table S1.

### 2.9. Protein Extraction and Western Blot

Cells and liver tissues were lysed in Radioimmunoprecipitation assay buffer supplemented with protease inhibitors. Protein concentrations were determined using the BCA protein assay (Thermo Fisher Scientific). Equal amounts of protein were resolved on 10–15% SDS-PAGE gels and transferred to PVDF membranes (0.45 µm, Millipore, Burlington, MA, USA). Protein bands were visualized using SuperSignal™ West Pico PLUS (Thermo Fisher Scientific) and detected with the iBright FL1500 Imaging System (Thermo Fisher Scientific). Densitometric analysis was performed using ImageJ (NIH, version 1.54g). Antibody details are provided in Supplementary Table S2.

### 2.10. Cytokine Array

Liver tissue lysates (150 µg protein) were analyzed using the Mouse XL Cytokine Array (R&D Systems, Minneapolis, MN, USA, ARY028) according to the manufacturer’s instructions. Chemiluminescent signals were captured on an iBright Imaging System and quantified using ImageJ version 1.54g (Wayne Rasband and contributors, National Institutes of Health, Bethesda, MD, USA) with background subtraction. Values were normalized to the internal reference spots on each membrane.

### 2.11. Glucose and Insulin Tolerance Tests

Glucose tolerance tests (GTTs) were performed following a 6 h fast. For GTTs, mice received intraperitoneal glucose (3 g/kg body weight) and blood glucose levels were measured at 0, 30, 60, 90, and 120 min using a GlucoDr Blood Glucose Test Strip (Hasuco, Seoul, Republic of Korea). For insulin tolerance tests (ITTs), mice were fasted for 6 h and injected intraperitoneally with human insulin (0.75 U/kg body weight); blood glucose was measured at 0, 30, 60, 90, and 120 min. The area under the curve (AUC) was calculated using the trapezoidal method.

### 2.12. Statistics

Data are presented as mean ± SEM. For in vivo studies, each group contained four mice (n = 4 per group). For in vitro studies, data represent at least three independent experiments performed in triplicate unless otherwise indicated. Statistical analyses were performed using GraphPad Prism version 10.4.1 (GraphPad Software, San Diego, CA, USA). Group comparisons were assessed by one-way or two-way ANOVA followed by Tukey’s post hoc test. *p* < 0.05 was considered statistically significant. In the figures, significance is indicated as * *p* < 0.05, ** *p* < 0.01, and *** *p* < 0.001.

## 3. Results

### 3.1. PCB138 Induces Liver Injury and Metabolic Alterations In Vivo

**Figure 1:**
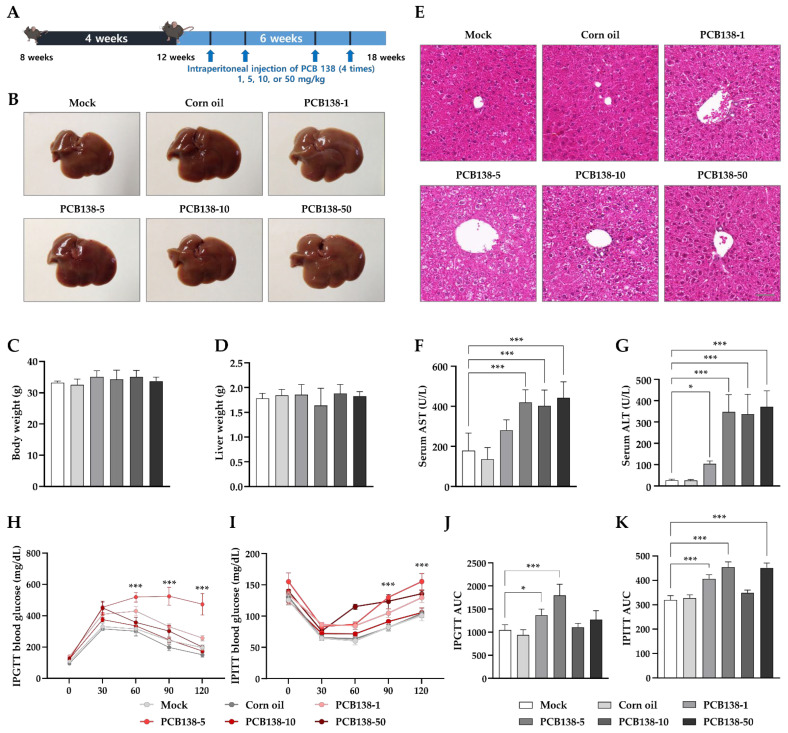
Effects of PCB138 on hepatic and metabolic parameters in mice: (**A**) Experimental design. Eight-week-old C57BL/6J mice received four intraperitoneal injections of vehicle (corn oil) or PCB138 (1, 5, 10, or 50 mg/kg) over six weeks (n = 4 per group). (**B**) Representative gross photographs of livers at sacrifice (18 weeks). (**C**) Body weight. (**D**) Liver weights. (**E**) Representative H&E-stained liver sections (scale bar = 50 μm). (**F**) Serum AST. (**G**) Serum ALT. (**H**) Intraperitoneal glucose tolerance test (IPGTT). (**I**) Intraperitoneal insulin tolerance test (IPITT). (**J**) AUC for IPGTT. (**K**) AUC for IPITT. Data are presented as mean ± SEM (n = 4). Statistical significance was determined by one-way ANOVA with Tukey’s post hoc test. * *p* < 0.05, *** *p* < 0.001 vs. vehicle.

C57BL/6J mice received four intraperitoneal injections of vehicle (corn oil) or PCB138 (1, 5, 10, or 50 mg/kg) over six weeks (Figure 1A). Gross liver morphology appeared comparable among groups (Figure 1B). Body weight at sacrifice and liver weight showed no significant differences among groups (Figure 1C,D). Histological examination revealed hepatocellular alterations in PCB138-exposed mice without steatosis (Figure 1E). Vehicle livers showed preserved lobular architecture with intact sinusoids, whereas PCB138 exposure produced progressive changes: mild sinusoidal dilatation and focal cytoplasmic alterations at 1 mg/kg; sinusoidal congestion, focal lobular disorganization, and hepatocellular ballooning at 5 mg/kg; hepatocellular swelling with eosinophilic cytoplasm, focal inflammatory infiltration, and sinusoidal congestion at 10 mg/kg; and widespread sinusoidal dilatation and congestion with focal hemorrhage and lobular disorganization at 50 mg/kg. Serum aspartate aminotransferase (AST) and alanine aminotransferase (ALT) were significantly elevated at 5, 10, and 50 mg/kg versus vehicle (Figure 1F,G). Metabolic assessments revealed differential effects on glucose homeostasis. Baseline fasting glucose (0 min) did not differ among groups. During the intraperitoneal glucose tolerance test (IPGTT), the 5 mg/kg group exhibited significantly higher blood glucose at 60, 90, and 120 min versus vehicle (Figure 1H). The area under the curve (AUC) confirmed impaired glucose tolerance at 1 mg/kg and 5 mg/kg versus vehicle (Figure 1J). In the intraperitoneal insulin tolerance test (IPITT), the 5 mg/kg group showed reduced insulin sensitivity, with significantly higher blood glucose at 90 and 120 min versus vehicle (Figure 1I). AUC values were elevated at 1, 5, and 50 mg/kg, whereas the 10 mg/kg group did not differ from vehicle (Figure 1K).

### 3.2. PCB138 Induces Hepatic Inflammation and Fibrosis In Vivo

**Figure 2:**
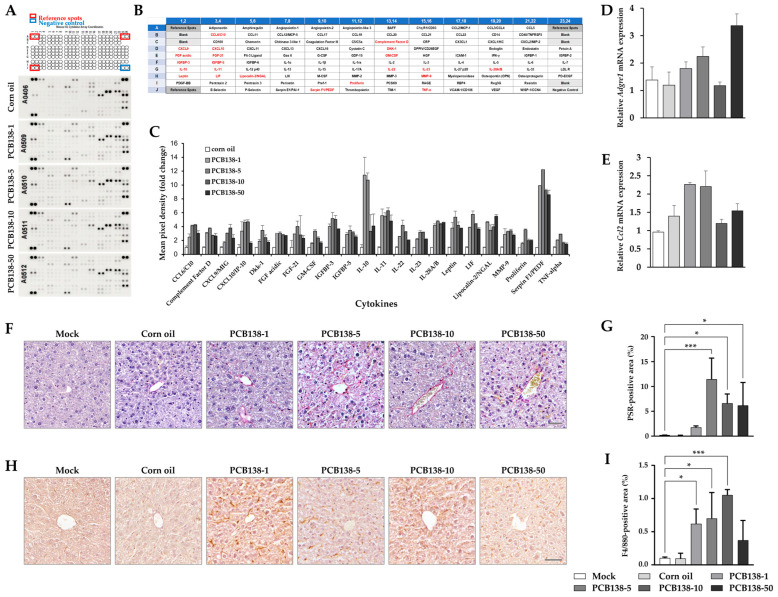
PCB138 increases hepatic cytokines, macrophage infiltration, and collagen deposition in vivo: (**A**) Cytokine array of liver tissue lysates after PCB138 treatment. (**B**) Array layout with reference, positive, and negative controls. (**C**) Quantification of cytokine levels. (**D**) Hepatic *Adgre1* (F4/80) mRNA expression. (**E**) Hepatic *Ccl2* (Mcp-1) mRNA expression. (**F**,**G**) Picrosirius Red staining of liver sections. Scale bar = 50 μm. (**H**,**I**) F4/80 immunohistochemistry of liver sections. Scale bar = 50 μm. Data = mean ± SEM (n = 4 per group). Statistical significance was determined using one-way ANOVA followed by Tukey’s post hoc test. * *p* < 0.05, *** *p* < 0.001 versus vehicle unless otherwise indicated.

Cytokine array analysis of liver tissue lysates showed that multiple inflammatory mediators were elevated in PCB138-treated mice compared with vehicle controls (Figure 2A–C). Among the 111 cytokines examined, notable increases were detected in IL-10, FGF-21, leptin, lipocalin-2, TNF-α, and MMP-9, indicating broad activation of inflammatory and metabolic signaling pathways. The qPCR analysis further confirmed these findings at the transcriptional level. Hepatic *Adgre1* (F4/80) mRNA expression was markedly elevated at 50 mg/kg (Figure 2D), whereas *Ccl2* (Mcp1) mRNA expression was notably increased at 1 and 5 mg/kg compared with vehicle controls (Figure 2E). Fibrotic alterations were assessed using Picrosirius Red staining. Collagen deposition was observed in all PCB138-treated groups relative to the vehicle group (Figure 2F). Quantitative analysis demonstrated that collagen-positive areas were significantly increased at 5, 10, and 50 mg/kg compared with vehicle controls (Figure 2G). F4/80 immunohistochemistry revealed increased macrophage infiltration in all PCB138 groups compared with the vehicle group (Figure 2H). Quantitative evaluation revealed significant increases in F4/80-positive cells at 1, 5, and 10 mg/kg relative to vehicle treatment (Figure 2I). Together, these results indicated that PCB138 exposure is associated with the upregulation of inflammatory mediators, enhanced macrophage recruitment, and increased hepatic collagen deposition in vivo.

### 3.3. PCB138 Induces Hepatic Iron Overload and Dysregulates Iron Metabolism

**Figure 3:**
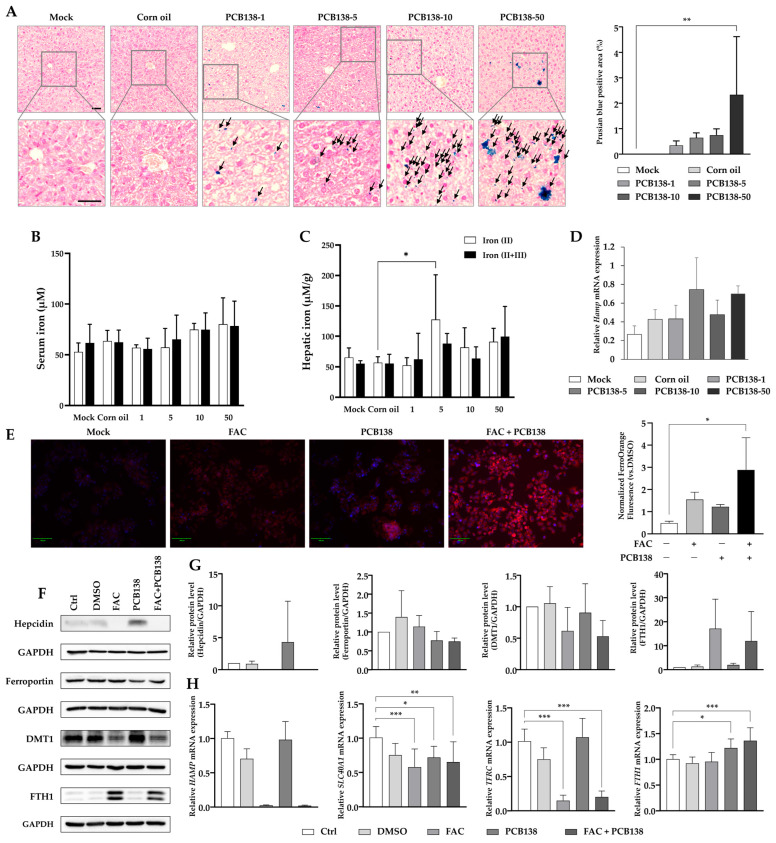
PCB138 induces hepatic iron accumulation and dysregulates iron metabolism genes in vivo and in vitro: (**A**) Prussian blue staining of liver sections showing iron deposition (arrows indicate regions of iron accumulation). Scale bar: 50 μm. (**B**) Serum iron levels in mice treated with vehicle or PCB138 (1, 5, 10, or 50 mg/kg). (**C**) Hepatic non-heme iron content, separated into ferrous iron [Fe(II)] and total iron [Fe(II + III)]. (**D**) Hepatic *Hamp* mRNA expression. (**E**) Intracellular Fe^2+^ levels in HepG2 cells detected by FerroOrange with Hoechst counterstaining (red indicates FerroOrange fluorescence for Fe^2+^; blue indicates nuclei stained with Hoechst 33342). Cells were treated with vehicle (DMSO), PCB138 (30 μM), ferric ammonium citrate (FAC, 200 μg/mL), or PCB138 + FAC for 24 h. Scale bar = 100 μm (**F**) Representative Western blot showing hepcidin, ferroportin, DMT1, and FTH1 protein expression with GAPDH as loading control. (**G**) Densitometric quantification of Western blot data. (**H**) Gene expression analysis of *HAMP* (hepcidin), *SLC40A1* (ferroportin), *TFRC* (transferrin receptor), and *FTH1* (ferritin heavy chain) mRNA in HepG2 cells. Data are presented as mean ± SEM (n = 4 for in vivo; n = 3 for in vitro). Statistical significance was determined by one-way ANOVA followed by Tukey’s post hoc test. * *p* < 0.05, ** *p* < 0.01, *** *p* < 0.001 versus vehicle unless otherwise indicated.

Prussian blue staining revealed hepatic iron deposition in PCB138-treated mice, with marked accumulation observed at 50 mg/kg compared to vehicle controls (Figure 3A). Serum iron levels showed alterations with PCB138 exposure (Figure 3B). Hepatic non-heme iron analysis demonstrated that ferrous iron [Fe(II)] was elevated in the 5 mg/kg group compared with vehicle (Figure 3C), while total iron [Fe(II + III)] remained unchanged. Hepatic *Hamp* mRNA expression was upregulated at 5 mg/kg and 50 mg/kg compared with vehicle (Figure 3D). In HepG2 cells, FerroOrange staining revealed that intracellular Fe^2+^ levels were highest in the combined PCB138 plus ferric ammonium citrate (FAC) treatment group (Figure 3E). Western blot analysis demonstrated that PCB138 treatment increased hepcidin protein expression and decreased ferroportin expression, with further reduction in the PCB138 + FAC group. DMT1 expression remained unchanged with PCB138 alone but decreased with FAC and PCB138 + FAC treatments. FTH1 expression increased with FAC and PCB138 + FAC treatments, while PCB138 alone showed no change (Figure 3F,G). Gene expression analysis in HepG2 cells revealed that *HAMP* mRNA increased with PCB138 treatment, while *SLC40A1* (ferroportin) mRNA decreased. *TFRC* mRNA expression was reduced by FAC and further suppressed by PCB138 + FAC, with no effect from PCB138 alone. *FTH1* mRNA expression increased with both PCB138 and PCB138 + FAC treatments (Figure 3H).

### 3.4. PCB138 Promotes Adiposity and Adipose Inflammation In Vivo

**Figure 4:**
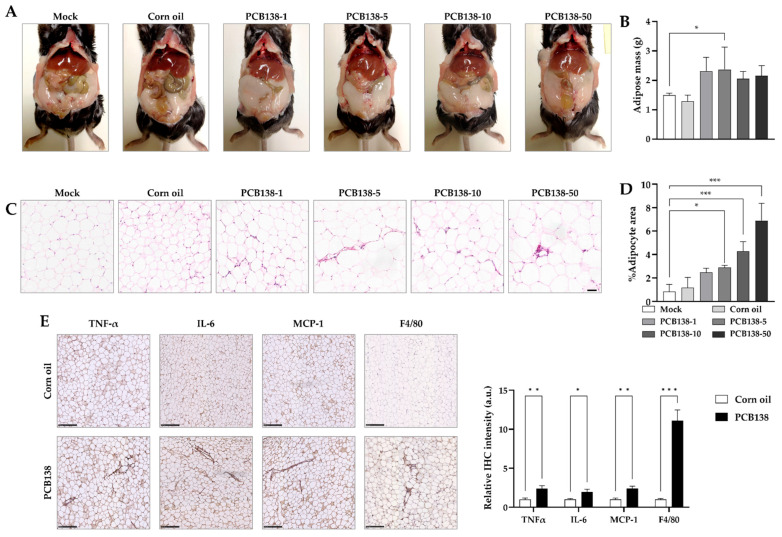
PCB138 increases visceral adiposity, induces adipocyte hypertrophy, and promotes pro-inflammatory cytokine expression in adipose tissue: (**A**) Representative gross images of mice after six weeks of PCB138 treatment (1, 5, 10, and 50 mg/kg, i.p.). (**B**) Quantification of visceral adipose tissue mass. (**C**) Representative H&E staining of epididymal white adipose tissue. Scale bar = 50 μm. (**D**) Quantification of adipocyte size from H&E staining using ImageJ version 1.54g. (**E**) Immunohistochemical staining of epididymal adipose tissue for TNF-α, IL-6, MCP-1, and F4/80. Scale bar = 250 μm. Statistical significance was determined using one-way ANOVA followed by Tukey’s post hoc test. * *p* < 0.05, ** *p* < 0.01, *** *p* < 0.001 versus vehicle control unless otherwise indicated. Data are presented as mean ± SEM; n = 4 per group.

Gross examination showed that PCB138-treated mice exhibited increased visceral fat compared with vehicle controls (Figure 4A). Quantitative analysis demonstrated a significant increase in total adipose tissue mass in PCB138-exposed groups (Figure 4B). Histological evaluation of epididymal white adipose tissue by H&E staining revealed hypertrophic adipocytes in PCB138-treated mice relative to the controls (Figure 4C). Image-based quantification confirmed a significant increase in adipocyte size following PCB138 exposure (Figure 4D). Immunohistochemical analysis further demonstrated increased expression of TNF-α, IL-6, MCP-1, and the macrophage marker F4/80 in adipose tissue (Figure 4E). Statistical analysis confirmed that all four markers were significantly elevated in PCB138-treated mice compared with the corn oil control (Figure 4E).

### 3.5. PCB138-Primed Adipocytes Drive Hepatocyte Iron Overload and Inflammatory Signaling

**Figure 5:**
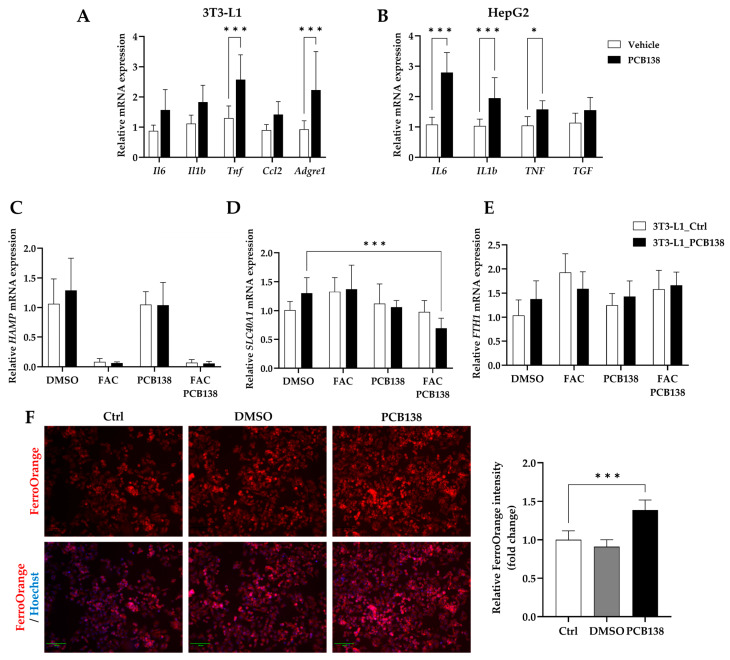
PCB138-primed adipocytes enhance hepatocyte iron overload and cytokine expression through adipocyte–hepatocyte cross-talk: (**A**) mRNA expression of IL-6, IL-1β, TNF-α, MCP-1, and F4/80 in differentiated 3T3-L1 adipocytes after PCB138 exposure. (**B**) mRNA expression of IL-6, IL-1β, TNF-α, and TGF-β in HepG2 hepatocytes co-cultured with control or PCB138-treated 3T3-L1 adipocytes. (**C**–**E**) qPCR analysis of Hamp (hepcidin), Slc40a1 (ferroportin), and Fth1 in HepG2 hepatocytes exposed to conditioned media from PCB138-treated adipocytes. (**F**) FerroOrange staining of HepG2 hepatocytes cultured with conditioned media from control, DMSO, or PCB138-treated 3T3-L1 adipocytes, followed by medium replacement with FAC + PCB138; quantification of intracellular iron by ImageJ version 1.54g (red represents FerroOrange fluorescence for Fe^2+^, while blue indicates nuclei stained with Hoechst 33342). Scale bar = 100 μm. Statistical significance was determined using one-way ANOVA followed by Tukey’s post hoc test. * *p* < 0.05, *** *p* < 0.001 versus vehicle or mock control unless otherwise indicated. Data are presented as mean ± SEM; n = 3 for in vitro experiments.

In 3T3-L1 adipocytes, PCB138 treatment increased the expression of *Il6*, *Il1b*, *Tnf*, *Ccl2*, and *Adgre1*, with significant induction of *Tnf* and *Adgre1* compared to controls (Figure 5A). HepG2 cells co-cultured with PCB138-primed adipocytes displayed increased expression of *IL6*, *IL1B*, *TNF*, and *TGFB1* compared with vehicle co-cultures (Figure 5B). Among these, *IL6*, *IL1B*, and *TNF* were significantly upregulated. qPCR analysis of HepG2 cells further demonstrated alterations in iron-handling genes, including *Hamp* (Figure 5C), *Slc40a1* (ferroportin; Figure 5D), and *Fth1* (Figure 5E). Gene expression patterns varied depending on HepG2 treatment conditions (DMSO, FAC, or FAC + PCB138) and the priming status of 3T3-L1 adipocytes (control vs. PCB138). FerroOrange staining confirmed changes in intracellular Fe^2+^ in HepG2 cells (Figure 5F). HepG2 cells co-cultured with PCB138-primed adipocytes and exposed to FAC + PCB138 exhibited the highest FerroOrange fluorescence intensity. Quantification by ImageJ version 1.54g analysis showed significantly increased Fe^2+^ levels under these conditions compared with corresponding controls.

## 4. Discussion

This study established the iron–adipose axis as a novel pathogenic paradigm in environmental toxicant-induced liver injury [24,34]. PCB138 exposure produced TASH characterized by hepatic inflammation and fibrosis without steatosis [35]—a phenomenon we term “non-steatotic TASH.” This discovery challenges the conventional paradigm that requires hepatic steatosis for liver injury progression and highlights its critical implications for environmental health assessments [18].

PCB138 induced hepatocellular injury with features distinct from classical metabolic liver disease. Serum transaminases increased across multiple dose groups. Histology showed sinusoidal dilatation, hepatocellular swelling/ballooning, and lobular disorganization without steatosis. This non-monotonic pattern indicates that an intermediate dose (5 mg/kg) elicited a greater effect than higher doses, a response often seen with endocrine-disrupting chemicals that complicates risk assessment for persistent organic pollutants [17,36,37,38].

However, we did not formally model these non-monotonic trends in the present study. We recognize that techniques such as curve-fitting or benchmark dose modeling could quantitatively validate the non-linear dose–response relationship. This represents an opportunity for future analysis, as our sample size and dose spacing were limited. Future studies with more dose groups or combined data sets should apply formal non-linearity tests to confirm the significance of the observed inverted-U patterns [39,40].

The inflammatory and fibrotic responses occurred as primary events rather than secondary to steatosis. Cytokine array analysis revealed broad activation of inflammatory mediators, including IL-10, FGF-21, leptin, lipocalin-2, TNF-α, and MMP-9. Upregulation of *Adgre1* and *Ccl2* mRNA, coupled with histological evidence of F4/80-positive macrophage infiltration, indicates robust immune cell recruitment [41]. Picrosirius Red staining revealed significant collagen deposition, particularly at 5 mg/kg, indicating early fibrotic remodeling [42]. These findings suggested that PCB138 directly activated hepatic inflammatory pathways independent of lipid-mediated mechanisms [18,38].

PCB138 profoundly disrupted hepatic iron homeostasis through coordinated dysregulation of iron regulatory proteins. Prussian blue staining revealed hepatic iron deposition in PCB138-treated mice, consistent with toxicant-induced iron accumulation [43]. Biochemical quantification further demonstrated a non-monotonic pattern: ferrous iron [Fe^2+^] was significantly elevated in the 5 mg/kg group compared with corn oil, whereas total iron [Fe^2+^ + Fe^3+^] remained unchanged. This selective rise in Fe^2+^ highlighted the expansion of the labile, redox-active iron pool, which is capable of driving ROS production and sensitizing hepatocytes to ferroptotic injury [44]. At the molecular level, *HAMP* mRNA and hepcidin protein levels were significantly upregulated, whereas *SLC40A1* mRNA and ferroportin protein levels were suppressed, creating conditions that promoted hepatocellular iron retention [45,46]. Compensatory responses include suppression of *TFRC* and induction of *FTH1* mRNA, reflecting cellular attempts to buffer iron overload [47,48,49]. In HepG2 cells, PCB138 enhanced FAC-induced Fe^2+^ accumulation, demonstrating increased susceptibility to iron loading under toxicant exposure. Together, these data established iron dysregulation and labile Fe^2+^ expansion as previously unrecognized mechanisms of PCB-induced hepatotoxicity [18].

Adipose tissue underwent inflammatory transformation that paralleled hepatic injury [21,50]. PCB138 exposure increased visceral adiposity and induced adipocyte hypertrophy, accompanied by elevated expression of TNF-α, IL-6, MCP-1, and F4/80 in adipose tissue [50,51]. This adipose remodeling represents more than simple fat accumulation; it reflects transformation into a metabolically active, pro-inflammatory organ capable of systemic effects [52]. The simultaneous development of adipose inflammation and hepatic injury suggested that adipose-derived signals directly contributed to liver pathology through nonlipotoxic mechanisms [53].

Bidirectional hepatocyte-adipocyte crosstalk establishes the molecular basis for the iron–adipose axis. In 3T3-L1 adipocytes, PCB138 treatment significantly induced *Tnf* and *Adgre1* expression, with increases also observed in *Il6*, *Il1b*, and *Ccl2*. When co-cultured with HepG2 cells, PCB138-primed adipocytes enhanced hepatocyte expression of *IL6*, *IL1B*, and *TNF*, demonstrating adipose-to-liver inflammatory signaling [54]. Critically, these primed adipocytes exacerbated FAC + PCB138-induced Fe^2+^ accumulation in hepatocytes, with corresponding alterations in *HAMP*, *SLC40A1*, and *FTH1* expression patterns [37,38,46]. This adipocyte-mediated amplification of hepatocyte iron retention reveals a novel feed-forward mechanism, whereby adipose inflammation and hepatic iron dysregulation mutually reinforce each other [55].

These findings have significant public health implications for populations exposed to persistent organic pollutants. Current screening strategies focusing on hepatic steatosis may miss a substantial proportion of individuals with toxicant-induced liver injuries [56]. The candidate biomarkers identified in this study—particularly hepcidin, ferroportin, ferritin, transferrin saturation, and GLDH—offer potential tools for early detection and monitoring of environmental liver disease. The non-monotonic dose–response relationships observed highlight the inadequacy of traditional linear extrapolation models for the risk assessment of endocrine-disrupting chemicals, suggesting that environmental exposure standards may need to be reconsidered [57].

The iron–adipose axis framework suggests a novel therapeutic strategy for the treatment of environmental liver disease. Potential interventions include dual targeting of iron overload (iron chelation therapy, hepcidin antagonists, and ferroportin agonists) and adipose inflammation (anti-cytokine therapies and metabolic modulators) [17,22,58,59]. These approaches may extend beyond PCB138 to other environmental toxicants that disrupt iron homeostasis or promote adipose dysfunction, including other PCB congeners, organochlorine pesticides, and emerging contaminants. This mechanistic understanding will enable precision medicine strategies in which therapeutic interventions can be tailored based on specific disruptions in the iron–adipose axis [18,32,60].

Consistent with our hypothesis, previous studies have demonstrated that alleviating iron overload can mitigate liver injury. For instance, iron chelation or phlebotomy in murine NASH models reduces oxidative damage and fibrosis. Conversely, excessive iron exacerbates liver injury, and hepcidin induction has been identified as a key driver in toxicant-induced hepatic damage. These findings support a causal role for iron dysregulation in liver pathology [61,62].

While the PCB138 doses used here are supra-environmental, the resulting tissue burdens approximate those seen in lifetime human accumulation under high exposure scenarios. For example, PCB138 can accumulate in the low ppm range in rodent adipose tissue after high-dose exposure, whereas even highly exposed humans show serum PCB138 in the sub-ppm (ng/g lipid) range. This underscores that our dosing provides a worst-case internal dose for mechanistic insight [9,10].

Study limitations must be acknowledged. The intraperitoneal administration route and six-week exposure duration do not fully replicate chronic oral human exposure patterns typical of environmental contamination. The concentrations used in vitro exceed typical human serum PCB138 levels (0.5–50 ng/mL), necessitating physiologically based pharmacokinetic (PBPK) modeling to bridge experimental and environmental exposures [63].

Moreover, species-specific differences in iron metabolism and the reality of mixed pollutant exposures in environmental settings may affect translation to human populations.

Furthermore, the in vivo sample size (n = 4 per group) was relatively small. While this sample size was sufficient to detect large effect-size changes (e.g., several-fold increases in ALT, hepcidin, etc.), it may have limited our ability to detect more subtle differences, and results should be interpreted with caution. We acknowledge this as a study limitation and recommend confirming these findings in larger cohorts in future studies.

In addition, our study demonstrates a correlation between PCB138 exposure, hepcidin induction, and iron accumulation, but does not establish direct causation. We did not perform experiments with iron-modulating interventions (such as hepcidin gene knockdown, ferroportin stabilization, or iron chelators) to definitively prove that iron overload is the driving mechanism of hepatotoxicity. As a result, we cannot exclude the possibility that iron dysregulation is an associated epiphenomenon rather than a sole cause of liver injury. This remains to be tested in future studies.

Lastly, we did not directly assess lipid peroxidation (e.g., MDA, 4-HNE) or key ferroptosis-associated markers such as ACSL4 in this study. However, a preliminary Western blot analysis of GPX4, a central regulator of ferroptosis, revealed a mild, non-significant decrease in the PCB138 + FAC group compared to controls. Although not definitive, this trend—when considered alongside hepatic iron overload—raises the possibility that ferroptosis may be involved in the observed liver injury. Future studies incorporating specific ferroptosis markers and lipid ROS measurements will be necessary to substantiate this hypothesis.

Future studies should validate and extend the iron–adipose axis paradigm. Epidemiological studies examining iron metabolism markers and adipose-derived cytokines in PCB-exposed human cohorts are required to confirm their clinical relevance [64]. The investigation of other environmental toxicants within this framework will determine whether the iron–adipose axis represents a conserved mechanism of environmental hepatotoxicity [65]. Understanding how pollutant mixtures interact through these pathways is crucial for accurate risk assessment and development of comprehensive environmental health policies [66].

## 5. Conclusions

Our findings identified the iron–adipose axis as a novel mechanism driving non-steatotic TASH [27]. PCB138 disrupts hepatic iron metabolism [67] and amplifies adipose inflammatory signaling [68], thereby redefining the paradigm of toxicant-induced liver injury beyond steatosis [35,69]. This mechanistic insight highlights opportunities for improved biomarkers and therapeutic strategies to mitigate environmental hepatotoxicity.

## Data Availability

The data supporting this study, including raw ImageJ densitometry tables and source data for the figures, are available from the corresponding author on reasonable request. Original, uncropped Western blots have been provided to the journal as source data.

## Supplementary Materials

The following supporting information can be downloaded at https://www.mdpi.com/article/10.3390/toxics13110932/s1. Table S1: Primer sequences used for quantitative real-time PCR analysis; Table S2: Antibodies used for Western blot analysis.
