# Supplementary material for: Polychlorinated Biphenyl 138 Induces Toxicant-Associated Steatohepatitis via Hepatic Iron Overload and Adipose Inflammation
Source: Toxics. 2025 Oct 30;13(11):932. doi: 10.3390/toxics13110932 (PMC12656693; doi:10.3390/toxics13110932)
Supplement: Supplementary file 1 [file toxics-13-00932-s001.zip › toxics-3921031-supplementary.pdf]

Table S1. Primer sequences used for quantitative real-time PCR analysis.

		Sense (5′-3′)	Antisense (5′-3′)	Expected amplicon size (bp)
Mouse	***F4/80***	TGTGTCGTGCTGTTCAGAACC	AGGAATCCCGCAATGATG	139
	Mcp-1	GCTGGAGAGCTACAAGAGGATCA	ACAGACCTCTCTCTTGAGCTTGGT	85
	Tnf	GGTGCCTATGTCTCAGCCTCTT	GCCATAGAACTGATGAGAGGGAG	139
	Il6	TACCACTTCACAAGTCGGAGGC	CTGCAAGTGCATCATCGTTGTTC	116
	Il1b	TGGACCTTCCAGGATGAGGACA	GTTCATCTCGGAGCCTGTAGTG	148
	Gapdh	AGGTCGGTGTGAACGGATTTG	GGGGTCGTTGATGGCAACA	95
Human	***TGFβ***	TACCTGAACCCGTGTTGCTCTC	GTTGCTGAGGTATCGCCAGGAA	122
	TNF	CTCTTCTGCCTGCTGCACTTTG	ATGGGCTACAGGCTTGTCACTC	135
	IL6	AGACAGCCACTCACCTCTTCAG	TTCTGCCAGTGCCTCTTTGCTG	131
	IL1β	CCACAGACCTTCCAGGAGAATG	GTGCAGTTCAGTGATCGTACAGG	131
	FTH1	TGAAGCTGCAGAACCAACGAGG	GCACACTCCATTGCATTCAGCC	100
	TFRC	ATCGGTTGGTGCCACTGAATGG	ACAACAGTGGGCTGGCAGAAAC	131
	SLC40A1	GAGACAAGTCCTGAATCTGTGCC	TTCTTGCAGCAACTGTGTCACAG	117
	hepcidin	CTGACCAGTGGCTCTGTTTTCC	AAGTGGGTGTCTCGCCTCCTTC	128
	GAPDH	GTGGTCTCCTCTGACTTCAAC	TCTCTTCCTGTGCTCTTG	212

Table S2. Antibodies used for Western blot analysis.

Target protein	Company	Catalog number	Dilution	MW (kDa)
Hepcidin	Cell signaling technology	29572	1:1000	10
Ferroportin	Novus	NBP1-21502	1:1000	~ 62
DMT1	Cell signaling technology	15083	1:1000	55, 70 - 100
FTH1	Cell signaling technology	4393	1:1000	21
GAPDH	Santa Cruz	sc-32233	1:1000	37
